# Radio-opaque contrast agents for liver cancer targeting with KIM during radiation therapy (ROCK-RT): an observational feasibility study

**DOI:** 10.1186/s13014-024-02524-4

**Published:** 2024-10-08

**Authors:** Natalie Plant, Adam Mylonas, Chandrima Sengupta, Doan Trang Nguyen, Shona Silvester, David Pryor, Peter Greer, Yoo Young (Dominique) Lee, Prabhakar Ramachandran, Venkatakrishnan Seshadri, Yuvnik Trada, Richard Khor, Tim Wang, Nicholas Hardcastle, Paul Keall

**Affiliations:** 1 Image X Institute, University of Sydney, Suite 201, Biomedical Building (C81), 1 Central Ave, Eveleigh, NSW 2015 Australia; 2 Department of Radiation Oncology, Princess Alexandra Hospital, Brisbane, QLD Australia; 3 Department of Radiation Oncology, Calvary Mater Newcastle, Newcastle, NSW Australia; 4 Olivia Newton-John Cancer Wellness & Research Centre, Austin Health, Melbourne, VIC Australia; 5 Department of Radiation Oncology, Crown Princess Mary Cancer Centre, Sydney, NSW Australia; 6 Peter MacCallum Cancer Centre, Melbourne, VIC Australia

**Keywords:** Neoplasms, Radiotherapy, Kilovoltage Intrafraction Monitoring, Image Guidance, Stereotactic ablative Radiation Therapy (SABR), Liver, Radio-opaque contrast, Transarterial chemoemobilisation (TACE)

## Abstract

**Background:**

This observational study aims to establish the feasibility of using x-ray images of radio-opaque chemoembolisation deposits in patients as a method for real-time image-guided radiation therapy of hepatocellular carcinoma.

**Methods:**

This study will recruit 50 hepatocellular carcinoma patients who have had or will have stereotactic ablative radiation therapy and have had transarterial chemoembolisation with a radio-opaque agent. X-ray and computed tomography images of the patients will be analysed retrospectively. Additionally, a deep learning method for real-time motion tracking will be developed. We hypothesise that: (i) deep learning software can be developed that will successfully track the contrast agent mass on two thirds of cone beam computed tomography (CBCT) projection and intra-treatment images (ii), the mean and standard deviation (mm) difference in the location of the mass between ground truth and deep learning detection are ≤ 2 mm and ≤ 3 mm respectively and (iii) statistical modelling of study data will predict tracking success in 85% of trial participants.

**Discussion:**

Developing a real-time tracking method will enable increased targeting accuracy, without the need for additional invasive procedures to implant fiducial markers.

**Trial registration:**

Registered to ClinicalTrials.gov (NCT05169177) 12th October 2021.

## Background

Liver cancer is a global health concern with increasing incidence worldwide [1–3]. It is the sixth most common cancer globally and the third leading cause of cancer-related deaths [1]. Hepatocellular carcinoma (HCC) is the most common form of primary liver cancer accounting for ~ 85% of cases [1]. In Australia, the incidence and mortality rate of liver cancer has increased more than any other cancer [4].

Stereotactic Ablative Radiation Therapy (SABR) is a technique used to deliver high-precision, ablative doses of radiation in a small number of fractions to an extra-cranial target [5]. It is utilised to treat a variety of malignancies including in the lung [6], liver [7] and spine [8].

One of the challenges of radiation therapy (RT) is the significant movement of tumours that can occur during treatment, particularly for tumours in the liver due to respiratory motion [9]. SABR is often utilised as an alternative to surgery or thermal ablation in patients with limited functional liver reserve or where tumours lie close to bowel, pericardium, or central biliary structures. If respiratory motion is not accounted for, there can be increased dose to surrounding structures and subsequent injury. Therefore, the delivery of a precise ablative dose is highly dependent on being able to verify the tumour position and any associated movement [10]. Real-time image-guided radiation therapy (IGRT) is a targeting method used to track the movement of tumour tissue during radiation therapy by tracking the motion of fiducial markers implanted in or near the tumour tissue [11]. One of these technologies, Kilovoltage Intrafraction Monitoring (KIM), has recently been applied to liver SABR [12]. The markers are implanted by an invasive procedure which can be associated with a risk of bleeding, infection, marker migration, additional treatment delays and may require a general anaesthetic [13, 14]. The current study seeks to develop and investigate a deep learning method that maintains the real-time tracking benefits of KIM, without the need to implant markers into patients.

In HCC patients, chemotherapy is often delivered via transarterial chemoemobilisation (TACE), which involves the injection of chemotherapy drugs into the artery supplying the tumour combined with embolic particles to restrict blood supply and retain the therapeutic agent within the treatment zone, and a radio-opaque contrast agent. Radio-opaque contrast material within the tumour allows direct visualisation of the treated tumour at the time of TACE and is often retained in the tumour and visible on imaging for many months afterwards. The approach of treating HCC using TACE immediately before SABR is increasingly used, and there is the potential to utilise this retained radio-opaque contrast as a marker for non-invasive, real-time tracking of tumour motion [15–17].

The most common radio-opaque agent used in this setting is ethiodised oil, however, there are also some drug-eluting bead (DEB-TACE) formulations that incorporate iodine directly into the bead structure. These retained radio-opaque contrast agents are currently often used as a surrogate tumour marker to aid target verification before and during SABR.

This observational study aims to establish the feasibility of using residual radio-opaque contrast agent in patient images as a real-time guidance method for IGRT treatment of HCC by applying deep-learning to x-ray images obtained as standard of care during radiation therapy. Completion of this observational study will lead to a prospective use of real-time markerless KIM real-time IGRT for eligible liver SABR patients.

## Methods/design

### Aim

**Fig. 1:**
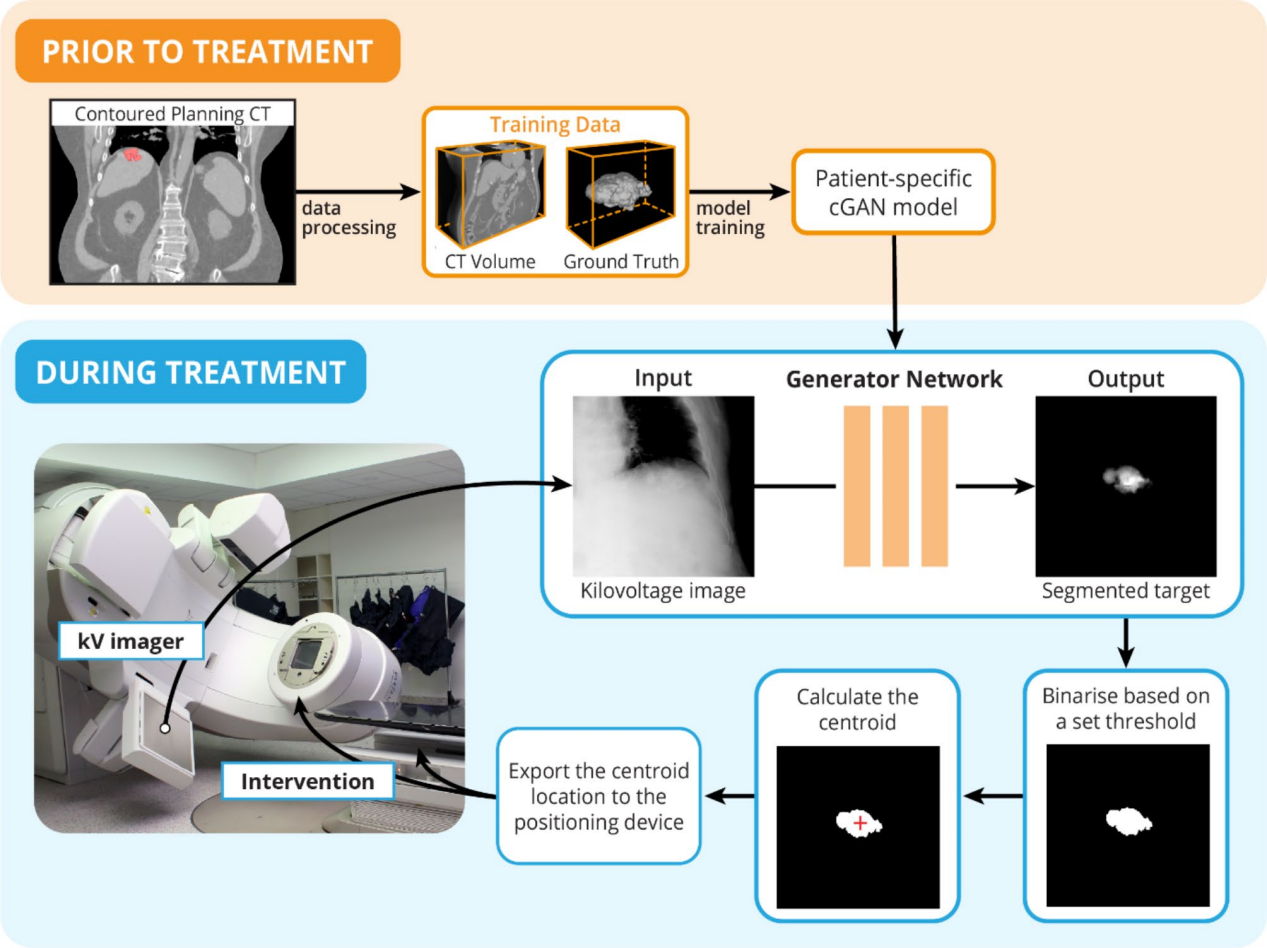
The clinical workflow for automatic target tracking using residual contrast agent is comprised of two key components: prior to treatment and during treatment. A patient-specific network is trained prior to the patient’s treatment using radiation therapy planning data. The generator network from the conditional generative adversarial network (cGAN) is used during the treatment to segment the target. The location of the segmented target can be used for motion management

This study aims to provide a non-invasive alternative to implanting fiducial markers to track tumour movement in real-time during SABR treatment of HCC. To determine whether radio-opaque contrast agents in the radiological images used to guide HCC radiotherapy can be used for real-time tracking of tumour movement, this study will train a deep-learning model to segment residual radio-opaque agents in radiation therapy planning images then attempt to accurately detect the agents in images obtained during treatment, for use with motion management software (KIM). The deep learning real-time tracking process is shown in Fig. 1.

### Design

**Fig. 2:**
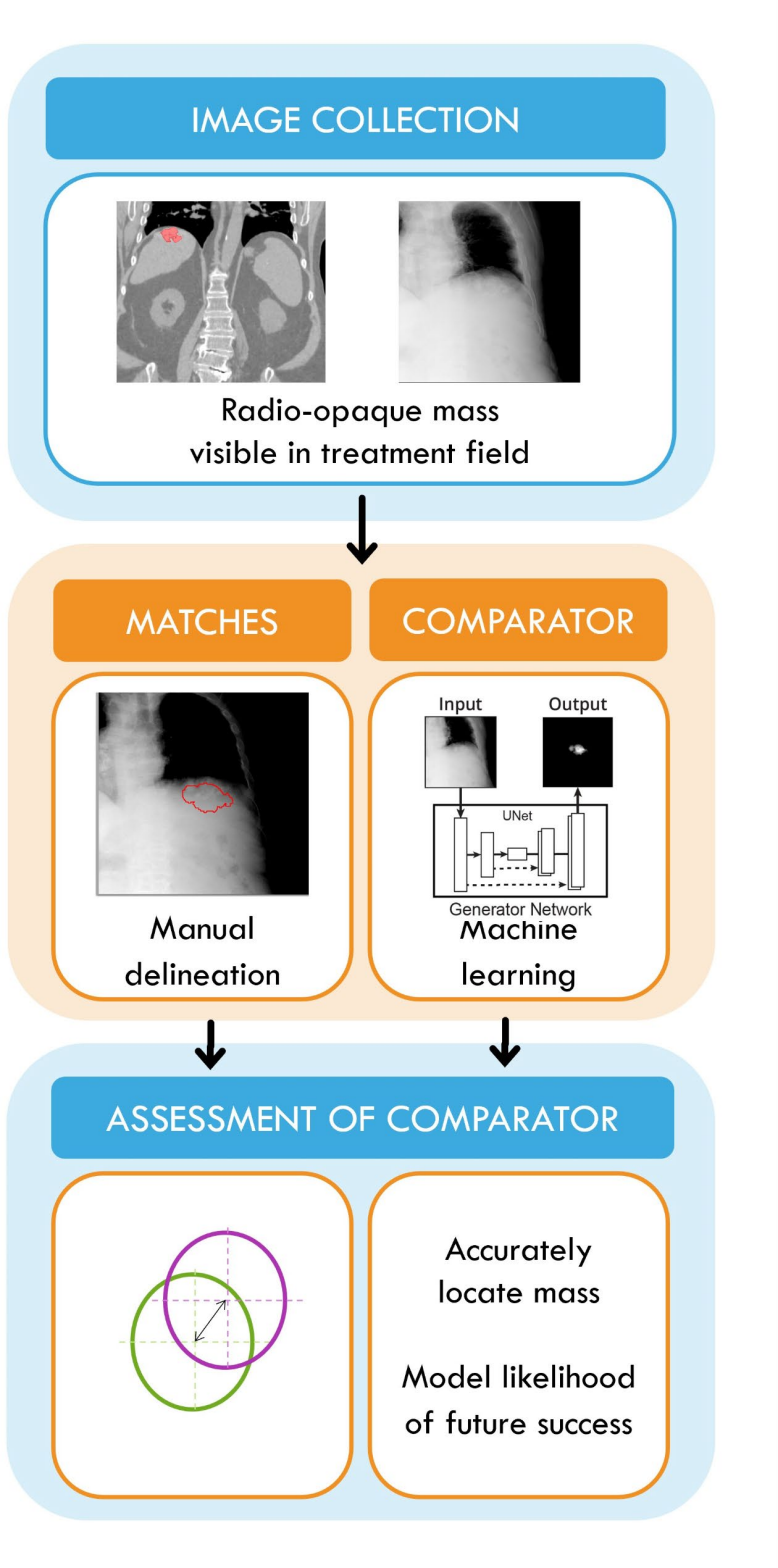
Study Design. Standardly collected radiation therapy planning, CBCT projections and intra-treatment monitoring images will be used to created manually delineated matches. After improvement of existing mass-detecting software (KIM) through machine learning, the updated algorithm will be applied to un-delineated images to (i) detect the contrast mass, and (ii) locate its centre in comparison with manually delineated matches. If the contrast mass can be detected accurately, modelling for likelihood of KIM tracking success will be conducted

This observational study will recruit patients who had or will have SABR treatment for HCC following TACE chemotherapy. Standard of care radiotherapy planning and in-treatment x-ray images will be collected, and analysis will occur offline. Following deep-learning and KIM algorithm adaptation, the detected location of the contrast agent mass by KIM will be compared with manual delineation (Fig. 2).

### Hypotheses

We hypothesise that: (i) deep learning software can be developed that will successfully track the contrast agent mass on two thirds of cone beam computed tomography (CBCT) projection and intra-treatment images (ii), the mean and standard deviation (mm) difference in the location of the mass between ground truth and deep learning detection are ≤ 2 mm and ≤ 3 mm respectively and (iii) statistical modelling of study data will predict tracking success in 85% of trial participants.

### Eligibility criteria

This study will recruit 50 participants who: will be ≥ 18 years of age; had or will have SABR for HCC; will/have residual radio-opaque contrast (e.g., ethiodised oil or drug-eluting beads containing iodine) from prior TACE chemotherapy visible within the imaging field on RT planning CT scans; will provide written informed consent or meet criteria for a waiver of consent; and will have the minimum image dataset available in the required format.

### Participants

Participants will be recruited from four Australian sites that currently use SABR to treat primary liver cancer; Princess Alexandra Hospital in Queensland, Calvary Mater Newcastle Hospital and the Crown Princess Mary Cancer Centre in New South Wales, and the Austin Health in Victoria. The Image-X Institute at the University of Sydney will develop the KIM algorithm and software for ground truth delineation and provide central study coordination.

### Consent/recruitment

Participants who had their radiation therapy prior to site activation will be recruited retrospectively and a waiver of the need for consent will be sought from an approved HREC (Human Research Ethics Committee) and governing State Health Data Custodian. Participants are otherwise recruited prospectively using a HREC-approved patient information sheet and consent form prior to starting RT.

### Datasets

The minimum RT treatment dataset required from retrospectively recruited participants includes the 3D or 4D CT scans used for planning, contours, treatment plans and pre-treatment 3D or 4D CBCT reconstructed images in DICOM format, and 2D projection images. In-treatment x-ray images are mandatory for prospectively recruited participants and desirable from those retrospectively recruited and any additional images from screening or testing sessions are desirable but not mandatory from all participants.

In addition to RT treatment images, data will be collected on participant characteristics (demographics, medical history, diagnosis), TACE chemotherapy (type of contrast agent - ethiodised oil or drug eluting beads, date of procedure), and other RT treatment-related data (treatment centre and treating doctor, treatment device and version, imaging system type/model, motion management techniques such as free-breathing or breath hold, the use of abdominal compression, and breathing training).

### Matching

**Fig. 3:**
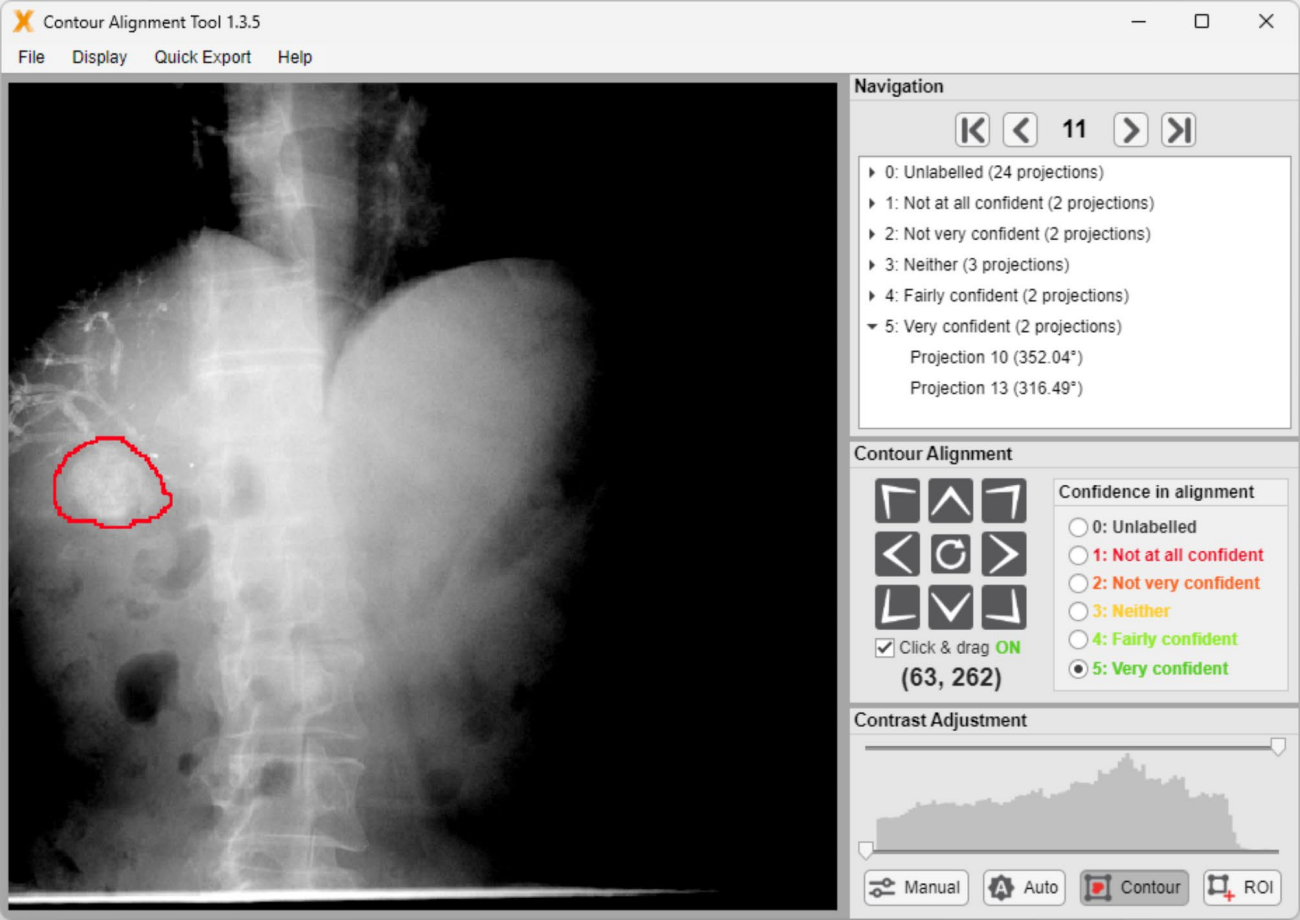
The contour alignment tool graphical user interface with an unaligned contour. The red contour (liver contrast agent mass) can be repositioned by the user

Manual delineation of the contrast mass on the planning CT and pre-treatment CBCT images (3D or 4D) with a purpose-designed alignment tool using MATLAB Runtime R2021a (version 9.10) (Fig. 3) will provide a ground truth location of the centre of the contrast mass to which delineation by the KIM software can be compared. If more than one contrast mass is visible on the planning CT and pre-treatment CBCT images, these will be separately delineated, and then the two volumes will be combined into a single contrast mass structure. To ensure at least 100 labelled images will be available for each participant, the contour alignment tool will choose 35 projections per CBCT from each fraction that are equally spaced angularly over the scanning arc to represent a range of angles. Where the contrast agent mass cannot be equivocally identified manually, these images will not be used as the ground truth. Users of the alignment tool will give a confidence score for the alignment using a five-level Likert scale ranging from ‘Not at all confident’ to ‘Very confident’.

### Comparator

The adapted KIM software will be run on un-delineated copies of the acquired data used to determine the ground truth location of the contrast agent. The KIM program will output the 2D and 3D position of the centre of the contrast agent mass. The KIM algorithm may be adjusted as more patient data is acquired, and the same version of the software will be run across all patients for the analysis.

### Data collection and transfer

An anonymisation tool will be applied to all images and datasets will be coded with participants’ unique Study ID before transfer. Image data will be uploaded to a data-sharing platform created and maintained by the University of Sydney for this study via a site-specific unique link to study folders. To quantity the interobserver error, the image data will be independently manually delineated by a separate study site.

All other data will be obtained from the participant’s medical record by delegated study personnel at the recruiting site and entered in a password-protected online database (REDCap 12.5.8, Vanderbilt University), coded by Study ID and year of birth.

### Bias

The study design minimises bias/errors due to sampling (baseline characteristics), matching (accuracy of comparator delineation) and assessments. Baseline characteristics to be considered in final analyses include (i) type, size, shape, density, and location of the contrast agent, (ii) type of imaging and treatment machine, (iii) time since TACE, (iv) participant demographics, and (v) treatment site. Accuracy of the ground truth (manual contrast mass delineation on images) will be maximised by (i) providing sites with the same purpose-designed software to conduct delineation of contrast mass location on images, (ii) for the same projections, performing ground truth delineation by an independent observer who is a member of the research team from another site, and (iii) assessment by qualified and experienced medical physicists or radiation therapists. Final assessment of the KIM software (comparator) will be conducted (i) on images that have not been marked with the ground truth, (ii) by site study personnel who have not seen images marked with ground truth, (iii) by study personnel who are qualified and experienced medical physicists.

### Outcome measures

(i) the proportion of CBCT projection and intra-treatment images in which the KIM software detects a contrast mass. (ii) the mean and standard deviation of the difference (mm) of location of the centre of the contrast mass detected on CBCT projection and intra-fraction images by KIM software compared with the ground truth in each of the horizontal and vertical directions. (iii) the mean and standard deviation of the centroid error between the segmentation and ground truth will be calculated, and DICE analysis will be performed to measure the similarity between the two delineation methods. Characteristics of the participants, chemotherapy, RT, and images, (e.g., treatment machine type, treatment site; contrast agent type, density, size, shape, and location; and patient size, age, cancer stage and sex), will be used to create a generalised linear model, or appropriate alternative, to identity univariate and multivariate patient or treatment features that contributed to the success or failure of the KIM tracking algorithm.

### Preliminary data

**Fig. 4:**
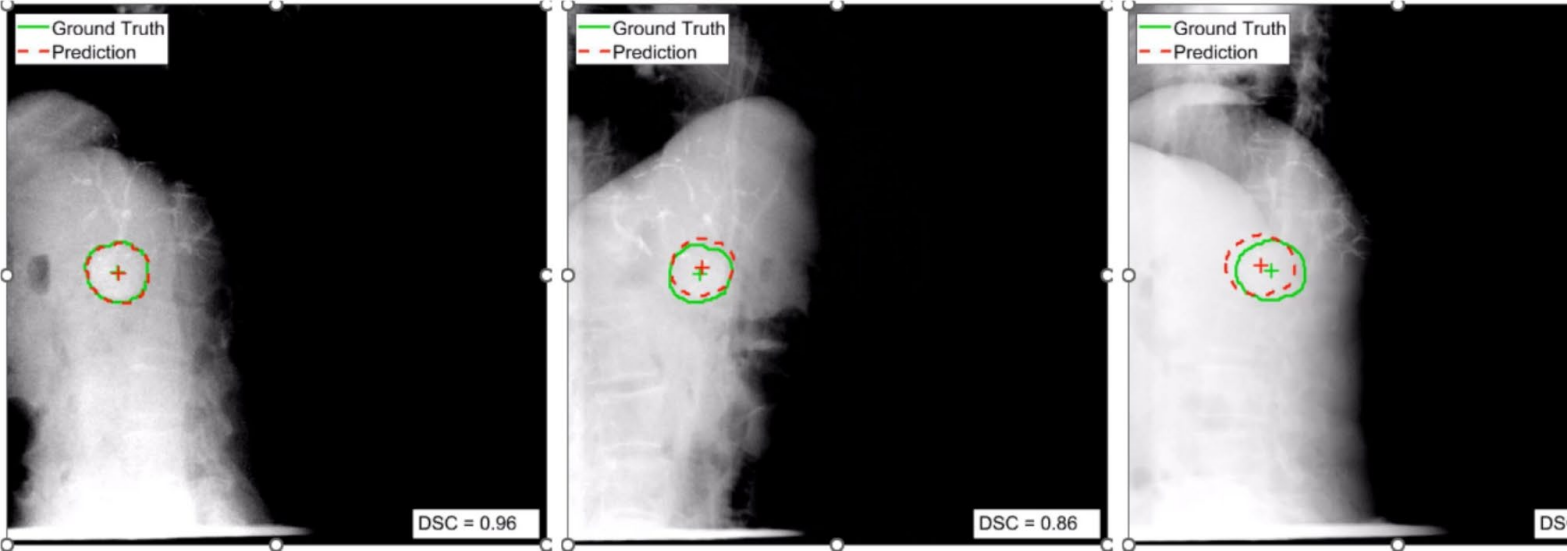
Examples of the target tracking prediction compared with the ground truth on kV images from different imaging angles from one fraction of one patient

**Fig. 5:**
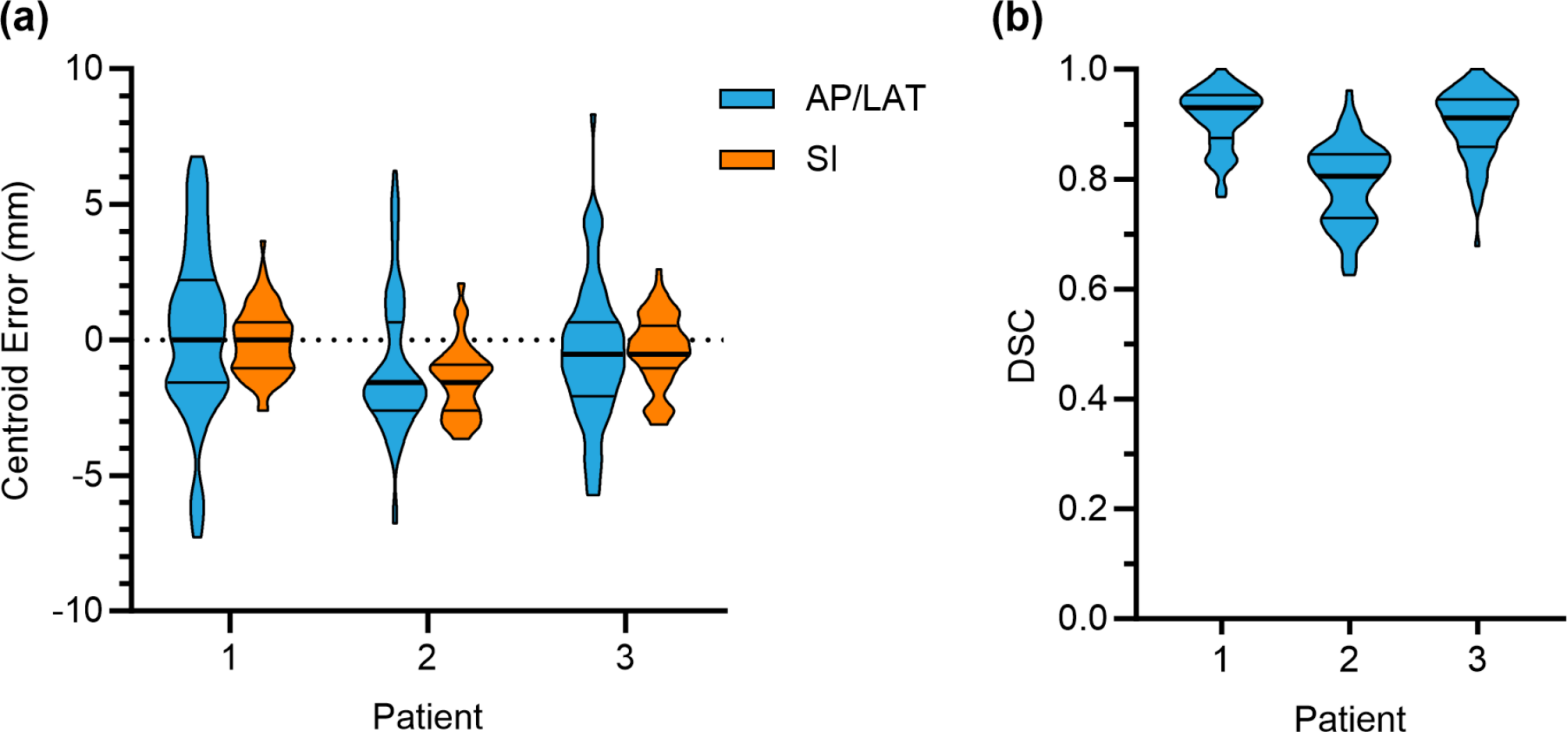
The centroid error and Dice similarity coefficient acquired from two fractions for three patients. AP = Anterior-Posterior; LAT = Lateral; SI = Superior-Inferior; DSC = Dice similarity coefficient

To investigate the feasibility of the ROCK-RT protocol, data from three patients recruited into ROCK-RT have been analysed. A conditional generative adversarial network [18] was used to train a patient-specific model to track the contrast mass from the pre-treatment data including data augmentation (translation and rotation). This model was then applied to the data acquired during treatment, representing the clinical scenario of real-time target tracking. Figure 4 shows examples of the target tracking prediction compared with the ground truth. Figure 5 quantifies the centroid error and Dice similarity coefficient acquired from two fractions for three patients using 35 images per fraction. The mean and standard deviation of the centroid tracking error in the anterior-posterior/lateral and super-inferior directions were − 0.4 ± 2.8 mm and − 0.6 ± 1.4 mm respectively. The mean Dice similarity coefficient was 0.87 ± 0.08.

## Discussion

The detection of residual radio-opaque contrast in the liver by KIM may lead to the development of technology that improves the accuracy, safety, and effectiveness of RT for liver cancer. The main challenges to this study are the availability of participants whose minimum image dataset is available, especially for retrospectively recruited participants, and the time commitment required by sites to contour images.

The hypotheses will be accepted if, in two thirds of participants (i), KIM detects the contrast mass; (ii) the mean and standard deviation (mm) difference in the location of the mass between ground truth and KIM detection are ≤ 2 mm and ≤ 3 mm, respectively and (iii) if a model of the success rate in the primary hypothesis and patient/treatment characteristics can predict KIM success in 85% of participants. Should the three hypotheses be accepted, there will be sufficient justification for the prospective use of KIM as a real-time image guided radiation therapy tool for liver cancer patients.

## Data Availability

No datasets were generated or analysed during the current study.

## Abbreviations

2D/3D/4D: Two-dimensional/three-dimensional/four-dimensional
CBCT: Cone beam computed tomography
DICE: Dice Similarity Coefficient
DICOM: Digital imaging and communications in medicine
HCC: Hepatocellular carcinoma
HREC: Human research ethics committee
IGRT: Image guided radiation therapy
KIM: Kilovoltage intrafraction monitoring
RT: Radiation therapy
SABR: Stereotactic ablative radiotherapy
TACE/DEB-TACE: Transarterial chemoemobilisation/Drug-eluting bead TACE

## References

[1] Sung H, Ferlay J, Siegel RL, Laversanne M, Soerjomataram I, Jemal A, et al. Global Cancer statistics 2020: GLOBOCAN estimates of incidence and Mortality Worldwide for 36 cancers in 185 countries. CA Cancer J Clin. 2021;71(3):209–49. 33538338 10.3322/caac.21660

[2] Llovet JM, Kelley RK, Villanueva A, Singal AG, Pikarsky E, Roayaie S, et al. Hepatocellular carcinoma. Nat Rev Dis Primers. 2021;7(1):6. 33479224 10.1038/s41572-020-00240-3 PMC13537433

[3] Villanueva A, Hepatocellular Carcinoma. N Engl J Med. 2019;380(15):1450–62. 30970190 10.1056/NEJMra1713263

[4] Cocker F, Chien Yee K, Palmer AJ, de Graaff B. Increasing incidence and mortality related to liver cancer in Australia: time to turn the tide. Aust N Z J Public Health. 2019;43(3):267–73. 30958629 10.1111/1753-6405.12889

[5] Chang BK, Timmerman RD. Stereotactic body radiation therapy: a comprehensive review. Am J Clin Oncol. 2007;30(6):637–44. 18091059 10.1097/COC.0b013e3180ca7cb1

[6] Timmerman R, Paulus R, Galvin J, Michalski J, Straube W, Bradley J, et al. Stereotactic body radiation therapy for inoperable early stage lung cancer. JAMA. 2010;303(11):1070–6. 20233825 10.1001/jama.2010.261 PMC2907644

[7] Hoyer M, Swaminath A, Bydder S, Lock M, Mendez Romero A, Kavanagh B, et al. Radiotherapy for liver metastases: a review of evidence. Int J Radiat Oncol Biol Phys. 2012;82(3):1047–57. 22284028 10.1016/j.ijrobp.2011.07.020

[8] Hall WA, Stapleford LJ, Hadjipanayis CG, Curran WJ, Crocker I, Shu HK. Stereotactic body radiosurgery for spinal metastatic disease: an evidence-based review. Int J Surg Oncol. 2011;2011:979214. 22312536 10.1155/2011/979214 PMC3263656

[9] Shirato H, Seppenwoolde Y, Kitamura K, Onimura R, Shimizu S. Intrafractional tumor motion: lung and liver. Semin Radiat Oncol. 2004;14(1):10–8. 14752729 10.1053/j.semradonc.2003.10.008

[10] Gargett M, Haddad C, Kneebone A, Booth JT, Hardcastle N. Clinical impact of removing respiratory motion during liver SABR. Radiat Oncol. 2019;14(1):93. 31159840 10.1186/s13014-019-1300-6 PMC6547575

[11] Keall PJ, Nguyen DT, O’Brien R, Caillet V, Hewson E, Poulsen PR, et al. The first clinical implementation of real-time image-guided adaptive radiotherapy using a standard linear accelerator. Radiother Oncol. 2018;127(1):6–11. 29428258 10.1016/j.radonc.2018.01.001

[12] Sengupta C, Nguyen DT, Moodie T, Mason D, Luo J, Causer T et al. The first clinical implementation of real-time 6 degree-of-freedom image-guided Radiotherapy for Liver SABR patients. Radiother Oncol. 2023:110031. 10.1016/j.radonc.2023.110031 38008417

[13] Dutta D, Kataki KJ, George S, Reddy SK, Sashidharan A, Kannan R, et al. Prospective evaluation of fiducial marker placement quality and toxicity in liver CyberKnife stereotactic body radiotherapy. Radiat Oncol J. 2020;38(4):253–61. 33249803 10.3857/roj.2020.00472 PMC7785839

[14] Bertholet J, Knopf A, Eiben B, McClelland J, Grimwood A, Harris E, et al. Real-time intrafraction motion monitoring in external beam radiotherapy. Phys Med Biol. 2019;64(15):15TR01. 31226704 10.1088/1361-6560/ab2ba8 PMC7655120

[15] Yue J, Sun X, Cai J, Yin FF, Yin Y, Zhu J, et al. Lipiodol: a potential direct surrogate for cone-beam computed tomography image guidance in radiotherapy of liver tumor. Int J Radiat Oncol Biol Phys. 2012;82(2):834–41. 21377291 10.1016/j.ijrobp.2010.12.050

[16] Buckstein M, Kim E, Ozbek U, Tabrizian P, Gunasekaran G, Facciuto M, et al. Combination Transarterial Chemoembolization and Stereotactic Body Radiation Therapy for Unresectable single large Hepatocellular Carcinoma: results from a prospective phase 2 trial. Int J Radiat Oncol Biol Phys. 2022;114(2):221–30. 35643250 10.1016/j.ijrobp.2022.05.021 PMC12951729

[17] Yoon SM, Ryoo BY, Lee SJ, Kim JH, Shin JH, An JH, et al. Efficacy and Safety of Transarterial Chemoembolization Plus External Beam Radiotherapy vs Sorafenib in Hepatocellular Carcinoma with Macroscopic Vascular Invasion: a Randomized Clinical Trial. JAMA Oncol. 2018;4(5):661–9. 29543938 10.1001/jamaoncol.2017.5847 PMC5885246

[18] Isola P, Zhu J-Y, Zhou T, Efros AA, editors. Image-to-image translation with conditional adversarial networks. Proceedings of the IEEE conference on computer vision and pattern recognition; 2017.

